# Effects of a fish oil containing lipid emulsion on plasma phospholipid fatty acids, inflammatory markers, and clinical outcomes in septic patients: a randomized, controlled clinical trial

**DOI:** 10.1186/cc8844

**Published:** 2010-01-19

**Authors:** Vera M Barbosa, Elizabeth A Miles, Conceição Calhau, Estevão Lafuente, Philip C Calder

**Affiliations:** 1 Institute of Human Nutrition, School of Medicine, University of Southampton, IDS Building, MP887 Southampton General Hospital, Tremona Road, Southampton, SO16 6YD, UK; 2 Hospital Padre Américo, Place of Tapadinha, Guilhufe, 4560-007 Penafiel, Portugal; 3 Department of Biochemistry, School of Medicine, Oporto University, Alameda Prof. Hernani Monteiro, Oporto, 4200 - 319 Porto, Portugal

## Abstract

**Introduction:**

The effect of parenteral fish oil in septic patients is not widely studied. This study investigated the effects of parenteral fish oil on plasma phospholipid fatty acids, inflammatory mediators, and clinical outcomes.

**Methods:**

Twenty-five patients with systemic inflammatory response syndrome or sepsis, and predicted to need parenteral nutrition were randomized to receive either a 50:50 mixture of medium-chain fatty acids and soybean oil or a 50:40:10 mixture of medium-chain fatty acids, soybean oil and fish oil. Parenteral nutrition was administrated continuously for five days from admission. Cytokines and eicosanoids were measured in plasma and in lipopolysaccharide-stimulated whole blood culture supernatants. Fatty acids were measured in plasma phosphatidylcholine.

**Results:**

Fish oil increased eicosapentaenoic acid in plasma phosphatidylcholine (*P *< 0.001). Plasma interleukin (IL)-6 concentration decreased significantly more, and IL-10 significantly less, in the fish oil group (both *P *< 0.001). At Day 6 the ratio PO_2_/FiO_2 _was significantly higher in the fish oil group (*P *= 0.047) and there were fewer patients with PO_2_/FiO_2 _<200 and <300 in the fish oil group (*P *= 0.001 and *P *= 0.015, respectively). Days of ventilation, length of intensive care unit (ICU) stay and mortality were not different between the two groups. The fish oil group tended to have a shorter length of hospital stay (22 ± 7 vs. 55 ± 16 days; *P *= 0.079) which became significant (28 ± 9 vs. 82 ± 19 days; *P *= 0.044) when only surviving patients were included.

**Conclusions:**

Inclusion of fish oil in parenteral nutrition provided to septic ICU patients increases plasma eicosapentaenoic acid, modifies inflammatory cytokine concentrations and improves gas exchange. These changes are associated with a tendency towards shorter length of hospital stay.

**Trials Registration:**

Clinical Trials Registration Number ISRCTN89432944

## Introduction

Sepsis results from a host inflammatory response to infection [1] and is characterised by high circulating concentrations of inflammatory cytokines such as tumor necrosis factor (TNF)-α, interleukin (IL)-1β, IL-6 and IL-8 [1,2]. Although conditions other than infections can trigger a state of hyperinflammation, sepsis requires special attention since even with current treatments it is often associated with very high mortality. Between the years 1979 and 2000, total sepsis-related mortality in the United States rose from 22 to 44 per 100,000 population [3], accounting for 9% of the overall annual mortality [4,5] with an enormous economic cost [6].

Septic patients receive the bulk of their nutrition by the parenteral route. Recently there has been increased interest in the lipid component of parenteral nutrition with the realisation that this not only supplies energy and essential building blocks, but may also provide molecules (that is, fatty acids) that are bioactive [7,8]. Traditionally used lipid emulsions are based solely upon soybean oil, which is rich in the n-6 fatty acid linoleic acid, or a 50:50 mix of vegetable oil rich in medium-chain saturated fatty acids and soybean oil (often termed MCT/LCT to indicate the mixture of medium chain and long chain triglycerides). More recently fish oil, which contains very long chain n-3 fatty acids, has been introduced into some lipid emulsions [9,10]. The rationale is partly that n-3 fatty acids act to reduce inflammatory responses [11], which may be promoted by an excessive or unbalanced supply of n-6 fatty acids. Compared with n-6 fatty acid rich vegetable oil, fish oil reduces the metabolic signs of endotoxemia in experimental animals [12], and lowers plasma cytokine concentrations [13] and improves survival [12,14]. Fish oil containing parenteral nutrition has been used in surgical patients demonstrating possible improvements in immune function [15,16] and reduced inflammation [16,17] which have been linked to a shorter stay in the intensive care unit (ICU) [16] and in hospital [16,18]. However there are few studies of fish oil containing lipid emulsions in septic patients in the ICU. Tappy et al. [19] demonstrated that parenteral fish oil is well tolerated and has only limited metabolic effects in critically ill patients, while Antebi et al. [20] showed that the use of fish oil in ICU patients requiring total parenteral nutrition may be associated with better liver function and improved antioxidant status. In two studies, Mayer et al. [21,22] reported diminished inflammation, including reduced TNF-α, IL-1β, IL-6, IL-8 and IL-10 production by cultured monocytes, in septic patients receiving a soybean oil-fish oil mix compared to those receiving soybean oil alone. These two studies did not report any clinical outcomes. Heller et al. [23] reported a dose-response effect of parenteral fish oil on antibiotic demand, length of hospital stay and mortality in critically ill patients. However, this latter study was not controlled. Recently, Friesecke et al. [24] reported that use of a mixed MCT/LCT/fish oil lipid emulsion in critically ill ICU patients had no effect on inflammatory markers, or on clinical outcomes including infections, ventilation requirement, or ICU or hospital stay compared with MCT/LCT. In contrast, use of fish oil in parenteral nutrition in severe pancreatitis patients resulted in a decreased inflammatory response, improved respiratory function and shortened Continuous Renal Replacement Therapy time [25]. Thus, there is only limited, and contradictory, information on the influence of fish oil containing parenteral nutrition in septic ICU patients on markers of inflammation and on clinical endpoints. However, studies of enteral nutrition providing fish oil, in addition to other potentially active ingredients, have demonstrated reduced inflammation, improved gas exchange and improved clinical outcome in patients with acute respiratory distress syndrome and/or acute lung injury [26-28].

This study was designed to investigate the potential benefits of using a parenteral lipid emulsion that includes fish oil in septic patients in the ICU. The outcomes were plasma phospholipid fatty acid profile, inflammatory mediators in plasma and produced by lipopolysaccharide-stimulated whole blood, routine biochemical and physiological markers, gas exchange and clinical outcomes. It was hypothesised that inclusion of fish oil would increase the n-3 fatty acid content of plasma phospholipids, would decrease circulating inflammatory cytokine concentrations and would reduce length of ICU and hospital stay.

## Materials and methods

### Study design

This study was a randomized, single blinded investigation of a parenteral lipid emulsion that contained fish oil in comparison with one that did not in patients admitted to a medical ICU with diagnosed sepsis. Patients were recruited from the ICU of Hospital Padre Américo, Penafiel, Portugal. The study was approved by the Ethics Committee *Comissão de Ética para a Saúde *from Hospital Padre Américo and was conducted in accordance with the Helsinki Declaration. Written informed consent was obtained from each patient's closest relative.

### Patient selection

Twenty-five patients with diagnosed systemic inflammatory response syndrome (SIRS) or sepsis [1] and who were predicted to need parenteral nutrition (severe pancreatitis, multiorgan failure, excisional surgery) were recruited at the time of admission to the ICU. Patients were recruited between March and December 2007. Sepsis was defined as suspected or proven infection plus SIRS (that is, presence of pyrexia, tachycardia, tachypnea and/or leukocytosis). Severe sepsis was defined as sepsis with organ dysfunction (hypotension, hypoxemia, oliguria, metabolic acidosis, and/or thrombocytopenia). Septic shock was defined as severe sepsis with hypotension despite adequate fluid resuscitation. Once identified as eligible to enter the study, patients were randomized by a sealed envelope to receive either a 50:50 (vol/vol) mixture of an oil rich in medium-chain fatty acids and soybean oil (termed MCT/LCT) (provided as a component of Nutriflex LipidSpecial^®^, B. Braun, Barcarena, Portugal) or a 50:40:10 (vol/vol/vol) mixture of an oil rich in medium-chain fatty acids, soybean oil and fish oil (termed fish oil) (provided as Lipolus^®^, B. Braun, Portugal). The principal differences are the presence of the long chain n-3 fatty acids eicosapentaenoic acid (EPA; 20:5n-3) and docosahexaenoic acid (DHA; 22:6n-3) in the fish oil containing emulsion where they contribute about 3.6% of fatty acids (2.5% of fatty acids as EPA and 1.1% of fatty acids as DHA) [29]. Nutriflex LipidSpecial is the routine means for supplying parenteral nutrition in Hospital Padre Américo ICU. Nutriflex LipidSpecial provides lipid (MCT/LCT emulsion), glucose and amino acids via a 1.25 liter three chamber bag. Lipoplus (250 ml) was added into 1 liter Nutriflex Special^® ^(B. Braun, Portugal) two chamber bags that provided glucose and amino acids. Nutriflex LipidSpecial had a lower glucose content than Nutriflex Special containing Lipoplus (144 g/l vs. 195 g/l), while the amino acid content was similar (57.4 g/l vs. 56 g/l). The amount of lipid contained within the final mixture was the same in both groups (40 g/l). Dipeptiven^® ^(Fresenius-Kabi, Carnaxide, Portugal) (50 ml/1250 ml bag) was included in both regimens. Both groups received electrolytes and vitamins.

Two of the 25 patients recruited did not start on parenteral nutrition and so are excluded from the study. Characteristics of the 23 patients who started on parenteral nutrition in the two groups are summarised in Table 1. From the 23 patients analysed, 13 received fish oil and 10 received MCT/LCT. Parenteral nutrition was administrated continuously over 24 hours, starting on the day after admission when the patient was hemodynamically stable, or if not, as soon as possible (Day 1 is defined as when parenteral nutrition was started). Blood samples were collected on admission, immediately prior to starting parenteral nutrition (that is, Day 1), 24 h after initiating parenteral nutrition (Day 2) and five days after initiating parenteral nutrition (Day 6). Blood was collected between 08:30 to 9:00 hours via an arterial line into ethylenediaminetetraacetic acid or lithium heparin.

**Table 1:** Characteristics of the patients in the two treatment groups

	**Fish oil group (n = 13)**	**MCT/LCT group (n = 10)**
	
Age range (years)	54 to 80	32 to 79
Age (years)	70 ± 2*	57 ± 5
Sex: male/female (n)	5/8	4/6
Height (m)	1.59 ± 0.1	1.63 ± 0.06
Weight (kg)	73.3 ± 18.01	76.8 ± 21.28
Body mass index (kg/m^2^)	28.9 ± 1.7	28.5 ± 2.6
Admitted from: Operating theatre/Emergency/Ward (n)	9/3/1	6/3/1
SAPS II	47.5 ± 5	41.6 ± 6.5
Sequential organ failure assessment score	9.5 ± 0.9	8.9 ± 1.2
Primary diagnosis: Sepsis/Severe sepsis/Septic shock (n)	8/4/1	5/2/3
Secondary Diagnosis: Cardiovascular/Respiratory/Renal/Gastric/Mental/Metabolic (n)	9/1/1/2/0/0	7/0/2/2/2/1

Data are shown for patients who received parenteral nutrition (n = 23).

SAPS - Simplified Acute Physiology Score; *P = 0.019 vs. MCT/LCT

Enteral nutrition was initiated as soon as possible, but for all patients this was beyond Day 6; enteral feeding was initiated as a mixed regimen with parenteral nutrition which used the same lipid emulsion as had been used for the study duration. For all patients the enteral feed used was Fresubin Original (Freseius-Kabi, Portugal); Fresubin Original contains fish oil and will provide 0.5 g of EPA plus DHA per 1,500 kcal.

### Nutritional assessment

Caloric intake was calculated using the Harris-Benedict [30] formula using a stress factor between 1.2 and 1.3. Weight was obtained at admission using a Hill-Rom^® ^bed (Hill-Rom Total Care, Batesville, IN, USA) which has a previously calibrated balance incorporated. Height was measured with the patient lying flat in bed.

### Routine laboratory measurements

Full blood count, biochemistry and coagulation were routinely assessed. Blood was centrifuged at 2,000 rpm for 15 minutes to obtain plasma which was stored at -70°C until analysis (within nine months).

### Whole blood culture and plasma collection

Whole blood was cultured essentially as described by Yaqoob et al. [31]. Whole blood was collected into lithium heparin and diluted 1:10 in Roswell Park Memorial Institute medium with 2 mmol/l L-glutamine and antibiotics (Sigma-Aldrich, Schnelldorf, Germany). The diluted blood was cultured in duplicate, with and without 10 μg/ml of *E. coli *0111:B4 lipopolysaccharide (LPS) (Sigma-Aldrich, Schnelldorf, Germany). Culture plates were incubated for 24 h in a 95% air 5% CO_2 _atmosphere and at 37°C. After this, the supernatant medium was collected and stored at -70°C until analysis (within nine months).

### Cytokine and eicosanoid analyses

Cytokines, prostaglandin (PG) E_2 _and leukotriene (LT) B_4 _were measured in plasma and cytokines and PGE_2 _in whole blood culture supernatants. Cytokines and eicosanoids were measured using enzyme-linked immunosorbent assays (ELISA) and following the manufacturer's instructions. IL-1β, IL-6, IL-10 and TNF-α ELISA kits were from Invitrogen (Paisley, UK), PGE_2 _ELISA kits from R&D Systems (Abingdon, UK) and LTB_4 _ELISA kits were from Cayman Chemical (Ann Arbor, MI, USA). Lower limits of detection were: IL-1β 0.06 pg/ml, IL-6 104 fg/ml, IL-10 0.2 pg/ml, TNF-α 0.09 pg/ml, PGE_2 _27.5 pg/ml, and LTB_4 _4 pg/ml.

### Fatty acid composition of plasma phosphatidylcholine

Fatty acid composition of plasma phospholipids (phosphatidylcholine; PC) was determined by gas chromatography as described [32].

### Statistical analysis

Data are presented as mean ± SEM, unless indicated otherwise. Statistical analyses were performed using SPSS version 14 (SPSS, Chicago, IL, USA). One factor ANOVA was used to analyse changes over time within a treatment group. Student's t-test was used for comparisons between time points and for comparisons between groups at a particular time point; equal variances were not assumed. Linear correlations were determined as Pearson's correlation coefficients. In all cases, a value of *P *< 0.05 was taken to indicate statistical significance.

## Results

### Energy and nutrient intakes

Energy, lipid, and amino acid intakes did not differ significantly between the groups (Table 2). However, glucose intake was significantly higher in the fish oil group (Table 2). The fish oil group received an average of 6.4 g/d of fish oil (Table 2), providing an average of 1.6 g EPA plus 0.7 g DHA/d (that is, 2.3 g long chain n-3 fatty acids/d).

**Table 2:** Energy and nutrient intake in the two treatment groups

	Fish oil group	MCT/LCT group
Energy intake (kcal/d)	2057 ± 418	1857 ± 255
(kcal/kg/d)	29.3 ± 7.6	25.3 ± 5.6
Amino acid intake^† ^(kcal/d)	329.1 ± 67.0	356.6 ± 50.2
(g/d)	82.3 ± 16.8	89.2 ± 12.6
(g/kg/d)	1.17 ± 0.30	1.22 ± 0.28
Glucose intake (kcal/d)	1151 ± 234**	909.8 ± 125.0
(g/d)	287.9 ± 58.6**	227.5 ± 31.3
(g/kg/d)	4.10 ± 1.07*	3.10 ± 0.69
Total lipid intake (kcal/d)	575.9 ± 117.3	594.2 ± 81.7
(g/d)	63.9 ± 13.0	66.0 ± 9.1
(g/kg/d)	0.91 ± 0.24	0.90 ± 0.20
Lipid intake as fish oil (kcal/d)	57.59 ± 11.7***	0
(g/d)	6.4 ± 1.3***	0
(g/kg/d)	0.09 ± 0.02***	0

Data are mean ± SEM; ^†^Excluding glutamine and alanine provided in dipeptiven

*P = 0.018; **P < 0.01; ***P < 0.001 vs. MCT/LCT

### Plasma phosphatidylcholine fatty acid composition

**Figure 1:**
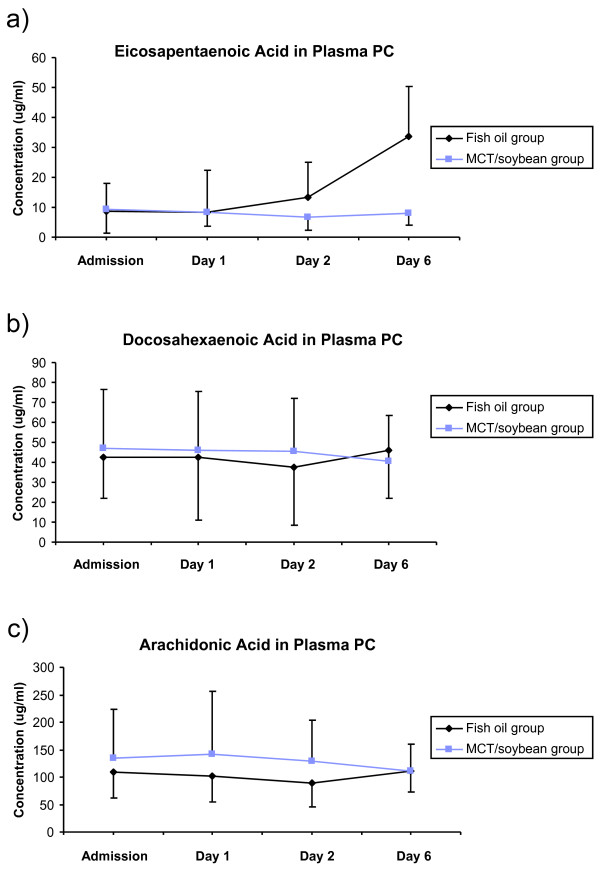
**The concentrations (μg/ml) of (a) EPA, (b) DHA and (c) arachidonic acid in plasma phosphatidylcholine in the two treatment groups**. *P < 0.001 vs. MCT/LCT at the same timepoint.

The fatty acid composition of plasma PC was measured as an indicator of n-6 and n-3 fatty acid status. Plasma PC contributes about 75% of plasma phospholipid [33] and functions as a transport pool of fatty acids delivering them to target tissues like leukocytes. The concentration of the long chain n-3 fatty acid EPA (20:5n-3) was increased in the fish oil group after supplementation such that levels were higher at Day 6 than at admission (*P *< 0.001), at Day 1 (p < 0.001) and at Day 2 (*P *= 0.003) (Figure 1a). EPA was higher in the fish oil group than in the MCT/LCT group at Day 6 (*P *< 0.001). The concentrations of DHA (22:6n-3) and of the long chain n-6 fatty acid arachidonic acid (20:4n-6) did not differ between the two groups (Figure 1b and 1c).

### Plasma cytokine and eicosanoid concentrations

Plasma cytokine, PGE_2 _and LTB_4 _concentrations did not differ between the two groups prior to initiation of parenteral nutrition (that is, Day 1) (Table 3). Linear regression demonstrated that both IL-1β and TNF-α decreased over time in both groups (IL-1β: *P *= 0.035 and *P *= 0.010 in the MCT/LCT and fish oil groups respectively; TNF-α: *P *= 0.036 and *P *= 0.005 in the MCT/LCT and fish oil groups respectively). Plasma IL-6 concentration also decreased over time in the fish oil group (*P *= 0.023). The changes in concentrations of IL-1β, IL-6, IL-10, and TNF-α between Day 6 and Day 1 were significantly different between groups when concentration at Day 1 was adjusted for; when concentration at Day 1, age and glucose supply were adjusted for; and when concentration at Day 1, age, glucose supply and simplified acute physiology score (SAPS) II at entry were adjusted for (all *P *< 0.001; Table 3). The decrease in IL-6 concentration was greater in the fish oil group while the decrease in IL-10 concentration was smaller in the fish oil group (Table 3). The decreases in IL-1β and TNF-α concentrations were similar between the groups, but were significantly smaller in the fish oil group (Table 3).

**Table 3:** Plasma cytokine and eicosanoid concentrations in the two treatment groups (pg/ml)

	Fish oil group		MCT/LCT group	
	
	Day 1	Day 6 - Day 1	Day 1	Day 6 - Day 1
IL-1β	5.0 ± 3.2	-3.8 ± 3.0*	5.7 ± 2.2	-4.2 ± 2.2
IL-6	8181 ± 5723	-4950 ± 6690*	1499 ± 732	-1242 ± 725
IL-10	44 ± 8	-29 ± 8*	85 ± 44	-64 ± 40
TNF-α	18.0 ± 3.1	-8.1 ± 3.6*	18.5 ± 5.1	-9.6 ± 4.9
PGE_2_	267 ± 126	391 ± 393	210 ± 134	513 ± 471
LTB_4_	338 ± 88	111 ± 147	421 ± 160	271 ± 148

Data are mean ± SEM

*P < 0.001 vs MCT/LCT group after adjusting for Day 1 value, or for Day 1 value, age and glucose supply, or for Day 1 value, age, glucose supply and SAPS II at entry.

### Cytokine and PGE_2 _production by LPS-stimulated whole blood cultures

Cytokine and PGE_2 _production by unstimulated and LPS-stimulated whole blood cultures did not differ between treatment groups at admission of patients or at any time point thereafter, even after controlling for age, glucose supply and SAPS II (data not shown). However, there was a significant effect of time, but not of treatment and there was no time × treatment interaction, on the LPS-stimulated production of TNF-α, IL-1β, IL-6 and IL-10 (two-factor ANOVA *P *= 0.002, 0.013, 0.001 and 0.008, respectively). Linear regression demonstrated that production of each of these cytokines increased with time, with a similar increase in both groups (Pearson's linear correlation coefficient = 0.394 (*P *< 0.001), 0.318 (*P *< 0.001), 0.416 (*P *< 0.001), 0.286 (*P *= 0.007) for TNF-α, IL-1β, IL-6 and IL-10, respectively).

### Routine laboratory measurements

There were no differences between the treatment groups with regard to blood leukocyte numbers, blood glucose concentration, C-reactive protein concentration, partial thrombin time, liver enzymes, and total bilirubin (Table 4). In the fish oil group blood monocyte numbers were significantly higher at Day 6 (1.41 ± 0.41 × 10^3^/μl) than at admission (0.52 ± 0.09 × 10^3^/μl; *P *= 0.017) and Day 2 (0.47 ± 0.11 × 10^3^/μl; *P *= 0.006). However, blood monocyte numbers did not differ between treatment groups at any time point. Fibrinogen concentration was significantly lower in the fish oil group at Day 2 (Table 4).

**Table 4:** Routine laboratory parameters in the two treatment groups

	Fish oil group				MCT/LCT group			
	
	Admission (n = 13)	Day 1 (n = 13)	Day 2 (n = 13)	Day 6 (n = 11)	Admission (n = 10)	Day 1 (n = 10)	Day 2 (n = 10)	Day 6 (n = 10)
Leucocytes (10^3^/μL)	14.6 ± 7.4	16.2 ± 9.2	14.2 ± 6.8	11.8 ± 5.4	17.7 ± 13.2	18.6 ± 12.8	15.2 ± 9.7	12.0 ± 6.0
Platelets (10^3^/μL)	212 ± 158	180 ± 163	126 ± 138	138 ± 122	215 ± 131	241 ± 122	204 ± 109	223 ± 150
Partial thrombin time (seconds)	46.2 ± 14.8	55.9 ± 21.5	66.5 ± 41.5	77.1 ± 94.5	38 ± 18.8	44.6 ± 20.7	40.6 ± 12.8	34.8 ± 6.6
Fibrinogen (mg/dL)	271 ± 136	286 ± 137	290 ± 159*	410 ± 94	428 ± 202	444 ± 184	481 ± 123	469 ± 76
Glucose (mg/dL)	149 ± 84	149 ± 53	206 ± 71	160 ± 35	139 ± 36	138 ± 48	177 ± 47	185 ± 69
CRP (mg/L)	177 ± 91	194 ± 110	215 ± 98	118 ± 53	182 ± 124	241 ± 105	239 ± 85	150 ± 108
AST (UI/L)	102 ± 99	86 ± 86	80 ± 64	48 ± 36	53 ± 41	51 ± 45	37 ± 33	37 ± 17
ALT (UI/L)	46.5 ± 51.4	40.3 ± 44.1	49.1 ± 52.8	45.9 ± 57.0	36.6 ± 29.7	32.2 ± 25.0	25.7 ± 20.6	77.0 ± 157.8
GGT (UI/L)	90.8 ± 107.9	89.5 ± 166.3	77.4 ± 134.2	129.9 ± 116.0	122.9 ± 120.0	92.3 ± 103.0	75.8 ± 75.2	103.4 ± 67.7
Bilirubin (mg/dL)	2.1 ± 0.7	2.4 ± 0.6	2.8 ± 0.8	3.0 ± 0.8	1.3 ± 0.4	1.3 ± 0.4	1.4 ± 0.3	1.6 ± 0.8

Data are mean ± SEM

CRP = C-reactive protein; AST = Aspartate transaminase; ALT = Alanine transaminase; GGT = γ-glutamyl transpeptidase

*P = 0.024 vs. MCT/LCT at the same timepoint

### Gas exchange

At Day 6, partial pressure carbon dioxide (PCO_2_) and the ratio partial pressure of oxygen/fraction of inspired oxygen (PO_2_/FiO_2_) were significantly higher in the fish oil group than in the MCT/LCT group (*P *= 0.033 and *P *= 0.047, respectively; Table 5), although the latter lost significance when age and glucose supply or age, glucose supply and SAPS II at entry were adjusted for. The proportions of patients with PO_2_/FiO_2 _<200 and <300 at Day 6 were significantly lower in the fish oil group than the MCT/LCT group (0% vs. 60% for <200 (*P *= 0.001; γ^2 ^test) and 36% vs. 70% for < 300 (*P *= 0.015; γ^2 ^test)). Conversely the proportion of patients with PO_2_/FiO_2 _>300 at Day 6 was significantly higher in the fish oil group than the MCT/LCT group (*P *= 0.015; γ^2 ^test). No other differences in gas exchange parameters were seen between the two groups (Table 5).

**Table 5:** Gas exchange parameters in the two treatment groups

	Fish oil group				MCT/LCT group			
	
	Admission (n = 13)	Day 1 (n = 13)	Day 2 (n = 13)	Day 6 (n = 11)	Admission (n = 10)	Day 1 (n = 10)	Day 2 (n = 10)	Day 6 (n = 10)
pH	7.27 ± 0.15	7.38 ± 0.11	7.41 ± 0.12	7.42 ± 0.06	7.37 ± 0.09	7.38 ± 0.11	7.44 ± 0.06	7.43 ± 0.1
Lactate (mmol/L)	3.2 ± 1.8	4.0 ± 1.7	4.5 ± 4.8	1.9 ± 0.7	2.7 ± 1.9	3.3 ± 1.9	2.4 ± 1.2	3.1 ± 2.7
PO_2 _(mm Hg)	198 ± 121	138 ± 45	127 ± 42	132 ± 44	178 ± 80	136 ± 42	145 ± 33	112 ± 38
PCO_2 _(mm Hg)	78 ± 125	39 ± 7	41 ± 6	48 ± 8*	36 ± 8	39 ± 10	40 ± 8	40 ± 8
PO_2_/FiO_2_	269 ± 125	248 ± 81	253 ± 102	331 ± 71**	262 ± 132	252 ± 125	299 ± 80	245 ± 107
PEEP (cm H_2_0)	5 (5, 7)	5 (5, 7)	5 (5, 7)	5 (5, 9)	5 (5, 6)	5 (5, 7)	5 (5, 6)	5 (5, 8)

Data are mean ± SEM, apart from PEEP values, which are median and interquartile range.

PO_2 _= partial pressure of oxygen; PCO_2 _= partial pressure of carbon dioxide; FiO_2 _= fraction of inspired oxygen; PEEP = positive end-expiratory pressure; *P = 0.033 vs. MCT/LCT group at the same timepoint (Student's t-test with equal variances not assumed; *P *= 0.016 after adjusting for age and glucose supply; *P *= 0.027 after adjusting for age, glucose supply and SAPS II at entry); **P = 0.047 vs. MCT/LCT group at the same timepoint (Student's t-test with equal variances not assumed; *P *= NS after adjusting for age and glucose supply or for age, glucose supply and SAPS II at entry).

### Clinical outcomes

Days of ventilation and length of stay in the ICU were not different between the two treatment groups (Table 6). The fish oil group tended to have a shorter length of hospital stay than the control group (22 ± 7 vs. 55 ± 16 days; *P *= 0.079; Table 6). This tendency remained when age and glucose supply were adjusted for (*P *= 0.062) and became significant when age, glucose supply and SAPS II at entry were adjusted for (*P *= 0.038). Three patients died within the course of the intervention (one in the MCT/LCT group and two in the fish oil group); all died from multiple organ failure. When data for these three patients who died within the first five days was excluded, length of stay remained shorter in the fish oil group, but the difference was not significant (*P *= 0.078 and *P *= 0.130 and 0.070 after adjustments; Table 6). A further five patients died after the completion of the intervention period but before Day 28 (three in the MCT/LCT group and two in the fish oil group). When data for these five patients were also excluded, length of stay was significantly shorter in the fish oil group (*P *= 0.044), although this significance was lost after adjustment for age and glucose supply (*P *= 0.068) or for age, glucose supply and SAPS II at entry (*P *= 0.057). Mortality was not different between groups, although 28 day mortality tended to be lower in the fish oil group (Table 6).

**Table 6:** Clinical outcomes in the two treatment groups

	Fish oil group (n = 13)	MCT/LCT group (n = 10)
Ventilated days	10 ± 4	11 ± 4
(excluding three patients who died in <5 days)	(11 ± 5)	(12 ± 4)
ICU stay (days)	12 ± 4	13 ± 4
(excluding three patients who died in <5 days)	(14 ± 5)	(14 ± 4)
Length of hospital stay (days)	22 ± 7*	55 ± 16
(excluding three patients who died in <5 days)	25 ± 8**	61 ± 17
(excluding all eight patients who died)	28 ± 9***	82 ± 19
five day mortality	15% (2 out of 13)	10% (1 out of 10)
28 day mortality	31% (4 out of 13)	40% (4 out of 10)

Data are mean ± SEM, apart from mortality values

*P = 0.079 vs. MCT/LCT (Student's t-test with equal variances not assumed; *P *= 0.062 after adjusting for age and glucose supply; *P *= 0.038 after adjusting for age, glucose supply and SAPS II at entry);

**P = 0.078 vs. MCT/LCT (Student's t-test with equal variances not assumed; *P *= 0.130 after adjusting for age and glucose supply; *P *= 0.070 after adjusting for age, glucose supply and SAPS II at entry);

***P = 0.044 vs. MCT/LCT (Student's t-test with equal variances not assumed; *P *= 0.068 after adjusting for age and glucose supply; *P *= 0.057 after adjusting for age, glucose supply and SAPS II at entry)

## Discussion

This study set out to evaluate the effects of a lipid emulsion containing a mixture of MCT, soybean oil and fish oil on plasma phospholipid fatty acid profile, inflammatory mediators in plasma and produced by LPS-stimulated whole blood, routine biochemical and physiological markers, gas exchange and clinical outcomes in septic patients in the ICU. The control group received a 50:50 mix of MCT and soybean oil. This is the first study of this fish oil containing lipid emulsion (that is, Lipoplus) in septic patients in the ICU, although it has been used previously in post-surgery patients [17-19,34,35]. In these latter patients, Lipoplus was found to decrease production or concentration of inflammatory eicosanoids [17,34] and cytokines [17] and to reduce length of hospital stay [18]. A different fish oil containing lipid emulsion (Omegaven) has also been used in post-surgery patients where it decreased production or concentration of inflammatory eicosanoids [36] and cytokines [16], improved immune function [15,16] and improved clinical outcomes [15,16,37]. Omegaven has also been used in septic patients [21,22], in critically ill ICU patients [24] and in patients with severe acute pancreatitis [25]. In some of these studies, use of Omegaven was associated with decreased inflammatory markers [21,22,25] and improved respiratory function [25]. Heller et al. [23] used Omegaven in a heterogeneous group of patients including post-surgical, septic and trauma patients and identified a dose-dependent reduction in mortality predicted from SAPS II score at entry. However, a recent study reported no effect of parenteral nutrition including Omegaven on inflammatory markers, or on clinical outcomes including infections, ventilation requirement, or ICU or hospital stay in critically ill ICU patients [24].

The current study found that five-day infusion of a MCT, soybean oil, fish oil mixture providing 6.4 g fish oil/day (equivalent to 2.3 g EPA plus DHA/d), increased EPA in the plasma phospholipid PC by an average of 3.8-fold, with no significant effect on DHA content and that this was associated with improved gas exchange and a tendency towards a shorter length of hospital stay. These are important findings since they indicate that the use of such an emulsion in this group of patients will improve clinical outcomes in comparison with what is seen with the more standard mix of MCT and soybean oil.

The increase in EPA content of PC is consistent with the recent report of a 2.4-fold increase in EPA in plasma phospholipids in healthy subjects receiving this same emulsion over a period of five days [29]. Likewise the lack of a significant change in either in DHA or arachidonic acid in plasma PC seen in the current study is consistent with what is reported by Simoens et al. [29]. These observations would suggest that any clinical benefit seen from the emulsion is due to EPA rather than DHA.

The tendency towards a reduction in length of hospital stay seen here (Table 6) was not a result of shorter ICU stay, and is consistent with findings in post-surgery patients receiving parenteral fish oil [15,16,18]. A previous study using a different lipid emulsion in ICU patients reports reduced ICU stay with higher fish oil administration [23] but this study was not controlled and relied upon historical data for comparison. Thus, this is the first randomised, controlled study reporting reduced length of hospital stay in septic ICU patients as a result of use of a fish oil containing lipid emulsion. The average dose of fish oil administered in the current study (6.4 g/day or 0.09 g/kg/d) is consistent with the dose that Heller et al. [23] found to be clinically favourable (>0.1 g/kg/d).

The current study identified a benefit of parenteral fish oil on gas exchange (Table 5). This is consistent with the recent report by Wang et al. [25] using parenteral fish oil in severe acute pancreatitis patients and with findings in acute respiratory distress syndrome patients receiving enteral fish oil [26]. The mechanism by which n-3 fatty acids improve respiratory function is not entirely clear, but recent work in the fat-1 mouse, which endogenously synthesizes n-3 fatty acids from dietary n-6 fatty acids, provides new information on this [38]. When exposed to LPS intratracheally, fat-1 mice showed reduced leukocyte invasion, protein leakage and inflammatory mediator (thromboxane B2, macrophage inflammatory protein-2) levels in lavage fluid compared with wild type mice. Furthermore ventilator compliance was improved in the fat-1 mice. This study shows a close link between anti-inflammatory effects of n-3 fatty acids, in this case seen at the level of the lung, and improved respiratory function.

Patients receiving parenteral fish oil showed more of a marked reduction in plasma IL-6 concentration than those in the MCT/LCT group and they also showed a smaller reduction in the anti-inflammatory cytokine IL-10. These findings concur with observations in post-surgery patients where plasma IL-6 concentrations were lower with parenteral fish oil [16,17]. These changes in plasma inflammatory markers may be part of the mechanism that explains the clinical benefits seen in this study. Differences in plasma TNF-α and IL-1β concentrations between the two groups were small, although significant.

In contrast to the effects on some plasma cytokines, parenteral fish oil did not affect LPS-stimulated production of inflammatory mediators from whole blood cultures. This contrasts with the observation of Mayer et al. [22] in septic ICU patients that LPS-stimulated production of inflammatory cytokines (TNF-α, IL-1β, IL-6, IL-8) by purified monocytes was lower in the fish oil group. However, the amount of fish oil and n-3 fatty acids used by Mayer et al. was much greater than the amount used in the current study (35 vs. 6.4 g fish oil/d; approximately 10 vs. 2.3 g EPA plus DHA/d). This may explain the difference in findings between the two studies.

In the current study the whole blood cultures responded well to LPS stimulation: the response to LPS increased with time in both groups. This is consistent with the recent observations of Kirchhoff et al. [39] who showed increased numbers of cytokine-positive monocytes following LPS stimulation of whole blood taken from patients with severe multiple injuries over the period 24 to 72 hours post-admission. Likewise, Heidecke et al. [40] demonstrated that in sepsis survivors there is an increase in LPS-stimulated production of IL-1β and IL-10 by monocytes over time. This recovery in cellular response appears to be associated with improved clinical outcome [39,40]. The observation that a poor inflammatory response of cultured cells taken early in sepsis is associated with poor patient outcome [39,40] seems to conflict with the many observations that a poor outcome is associated with higher concentrations of inflammatory cytokines in the circulation [41-43]. Thus there seems to be a miss-match between circulating pro- and anti-inflammatory cytokine concentrations which are elevated early in sepsis and the ability of leukocytes to produce pro- and anti-inflammatory cytokines which is impaired early in sepsis. Indeed, the current study indicates that, as plasma cytokine concentrations decline over time, the ability of leukocytes to produce those same cytokines when stimulated with LPS ex vivo increases. This seemingly paradoxical observation may be explained by considering the regulatory processes that occur to control inflammatory cytokine release. A strong inflammatory stimulus in vivo will lead to inflammatory cytokine production with an elevation in plasma cytokine concentrations. However, this will lead to negative feedback, for example inhibition of monocyte nuclear factor κB activation [44,45]. Therefore, upon restimulation ex vivo, the monocytes are less responsive [46]. Hence monocytes isolated from blood at a time when there is a high concentration of cytokines may show a low cytokine response when stimulated and vice versa.

The anti-inflammatory properties of n-3 fatty acids have been described and discussed in detail elsewhere [11,47,48]. The mechanisms involved include effects at the membrane level, on signal transduction pathways leading to transcription factor activation and altered patterns of gene expression, and on the pattern of lipid mediator generation. The discovery of resolvins generated from both EPA and DHA [49] has focussed attention on resolution of inflammation as a mechanism of action of n-3 fatty acids and on the differential effects of EPA and DHA on inflammatory processes. In the current study status of EPA, but not DHA, was increased in plasma PC, suggesting that the effects seen are due to EPA. EPA has been shown to decrease production of inflammatory eicosanoids and cytokines [see [11]] and is the precursor of inflammation resolving resolvin E1 [49]. Thus EPA may exert effects on both the generation of inflammatory mediators and on the resolution of inflammatory processes.

Whatever the mechanism(s) involved, this study demonstrates that a parenteral nutrition regimen including fish oil at the level used here does not impair the recovery of the ex vivo response of monocytes, but enhances the reduction in plasma IL-6 and diminishes the reduction in plasma IL-10 concentrations seen in the control group. Given that poor outcome is associated both with high plasma concentrations of inflammatory cytokines, including IL-6 [41-43] and with impaired ex vivo monocyte responses to LPS [39,40], the overall effects of fish oil seen in the current study appear to be of benefit.

Limitations of this study are its relatively small sample size, the difference in age between the two treatment groups (the average age of patients in the fish oil group was higher than in the MCT/LCT group), and the higher glucose supply in the fish oil group. However, despite the small sample size, significant effects on plasma phospholipid EPA content, plasma cytokines, and gas exchange parameters were observed. In order to account for the differences in age and glucose supply between the two groups, these were controlled for in statistical analysis of cytokines, gas exchange parameters and clinical outcomes. Even after accounting for the differences in age and glucose supply between the groups, effects of lipid emulsion on plasma cytokines and on gas exchange parameters remained significant and the trend for an effect on length of hospital stay was not altered.

## Conclusions

Inclusion of fish oil in parenteral nutrition provided to septic ICU patients increases plasma EPA status and this is associated with more marked changes in some cytokines in plasma, improved gas exchange and a trend towards reduced length of hospital stay.

## Key messages

• Including fish oil in the parenteral nutrition regimen received by septic ICU patients modified plasma IL-6 and IL-10 concentrations.

• Parenteral fish oil improved gas exchange in septic ICU patients.

• Parenteral fish oil decreased length of hospital stay in septic ICU patients.

## Abbreviations

ALT: Alanine transaminase; AST: Aspartate transaminase; CRP: C-reactive protein; DHA: docosahexaenoic acid; EPA: eicosapentaenoic acid; FiO_2_: fraction of inspired oxygen; GGT: γ-glutamyl transpeptidase; ICU: intensive care unit; IL: interleukin; LCT: soybean oil; LPS: lipopolysaccharide; LT: leukotriene; MCT: a triglyceride rich in medium-chain fatty acids; PC: phosphatidylcholine; PCO_2_: partial pressure of carbon dioxide; PEEP: positive end-expiratory pressure; PG: prostaglandin; PO_2_: partial pressure of oxygen; PO_2_/FiO_2_: ratio of the partial pressure of oxygen to the fraction of inspired oxygen; SAPS: simplified acute physiology score; SIRS: systemic inflammatory response syndrome; TNF: tumor necrosis factor

## Competing interests

PCC has received speaking honoraria from B. Braun, Fresenius-Kabi, Baxter Healthcare and Abbott Nutrition and has received research funding from B. Braun. The other authors declare that they have no competing interests.

## Authors' contributions

PCC and VMB designed the study. VMB recruited the patients, oversaw the intervention, collected the blood samples and collated the clinical data under the supervision of EL. VMB processed the blood samples and conducted the whole blood cultures under the supervision of CG. VMB and EAM conducted the ELISA assays. VMB conducted the fatty acid composition analysis under the supervision of PCC. VMB, EAM and PCC conducted the statistical analysis. VMB drafted the manuscript; EAM and PCC had significant input into finalising the manuscript.
